# Evaluation of Autologous Protein Solution Injection for Treatment of Superficial Digital Flexor Tendonitis in an Equine Model

**DOI:** 10.3389/fvets.2021.697551

**Published:** 2021-07-05

**Authors:** Angela M. Gaesser, Claire Underwood, Renata L. Linardi, Kayla M. Even, Virginia B. Reef, Snehal S. Shetye, Robert L. Mauck, William J. King, Julie B. Engiles, Kyla F. Ortved

**Affiliations:** ^1^Department of Clinical Studies, New Bolton Center, School of Veterinary Medicine, University of Pennsylvania, Kennett Square, PA, United States; ^2^McKay Orthopaedic Research Laboratory, Perelman School of Medicine, University of Pennsylvania, Philadelphia, PA, United States; ^3^Owl Manor, Warsaw, IN, United States; ^4^Department of Pathobiology, New Bolton Center, School of Veterinary Medicine, University of Pennyslvania, Philadelphia, PA, United States

**Keywords:** Pro-Stride, collagenase, regenerative, tendinopathy, tendonitis, orthobiologic, horse

## Abstract

Autologous protein solution (APS) has been used anecdotally for intralesional treatment of tendon and ligament injuries, however, its use in these injuries has never been studied *in vivo*. Our objective was to evaluate the effect of APS on tendon healing in an equine superficial digital flexor (SDF) tendonitis model. We hypothesized intralesional injection of APS would result in superior structural and biomechanical healing. SDF tendonitis was induced in both forelimbs of eight horses using collagenase injection. One forelimb was randomly assigned to receive an intralesional injection of APS, while the other was injected with saline. Ultrasonographic examinations were performed at weeks −1, 0, 2, 4, 8, and 12 following treatment. At 12 weeks, horses were euthanized and SDF samples harvested. Histologic evaluation, biomechanical testing, gene expression analysis, total glycosaminoglycan (GAG) and total DNA quantification were performed. Collagen type III (*COL3A1*) expression was significantly higher (*p* = 0.028) in saline treated tendon than in normal tendon. Otherwise, there were no significant differences in gene expression. There were no significant differences in histologic or ultrasonographic scores between groups. Mean total DNA content was significantly higher (*p* = 0.024) in saline treated tendons than normal tendons, whereas total DNA content was not significantly different between APS treated tendon and normal tendon. Elastic modulus was higher in APS treated than saline treated tendon, but the difference was not significant. Reduced expression of *COL3A1* in APS treated tendon may indicate superior healing. Increased total DNA content in saline treated tendon may indicate ongoing healing processes, vs. APS treated tendons which may be in the later stages of healing. Limitations include a relatively short study period and inconsistency in size and severity of induced lesions. Intralesional injection of APS resulted in some improvements in healing characteristics.

## Introduction

Tendon and ligament injuries are common in athletic horses, leading to decreased performance and often career-ending injuries. For example, superficial digital flexor (SDFT) tendonitis has a reported prevalence rate of 4–13% in racing thoroughbreds (1–4). Healing can be slow and repair tissue is often inferior in biomechanical strength and structure, therefore, highly susceptible to re-injury, with re-injury rates ranging from 42.8 to 53% (4, 5).

A variety of therapies have been employed for treating tendinopathies, including orthobiologics such as intralesional platelet rich plasma (PRP) and mesenchymal stem cell (MSC) injections. Several studies have shown that these therapies enhance endogenous tendon repair, resulting in superior biochemical, biomechanical, and histological healing (6–13). Treatment of naturally occurring tendonitis with MSCs has resulted in improved biomechanical and morphological characteristics (14), and decreased reinjury rate (8). In a collagenase induced tendonitis model, treatment with fetal derived embryonic like stem cells resulted in improved histological and ultrasonographic scores (15). In a recent study, treatment of surgically induced superficial digital flexor tendonitis with intralesional leukocyte-rich PRP resulted in an increased concentration of collagen and significantly higher load at failure with increased elasticity in comparison to lesions treated with saline (6). Another study showed that intralesional PRP treatment in a tendonitis model resulted in early increased neovascularization compared to controls, likely due to high concentrations of vascular endothelial growth factor (VEGF) within PRP (9).

Autologous protein solution (APS) can now be generated from an autologous stall-side dual device system (Pro-Stride™, Owl Manor, Warsaw, IN), offering an advantage over cell based therapies. This orthobiologic combines the beneficial effects of autologous conditioned serum (ACS), which contain increased levels of interleukin-1 receptor antagonist (IL-1ra) and interleukin-10 (IL-10), with the beneficial effects of PRP, which release a multitude of growth factors and anti-inflammatory cytokines. APS is also convenient because it does not require a 24 h incubation, as other ACS products require, and can be collected, processed, and ready to inject within 20 min (16).

The use of APS has been validated for the treatment of equine osteoarthritis in a recent study by Bertone et al. In this study, they found that APS contained a 5.8-fold increase in IL-1ra, a 3.6-fold increase in soluble tumor necrosis factor receptor 1 (sTNF-R), and a 3.4-fold increase in IL-10 compared with concentrations in blood (17). Similarly, Muir et al. found that APS contained a 6,500-fold increase in transforming growth factor beta (TGF-β) and a 3.3-fold increase in IL-1ra compared to plasma (18). Another study found that when chondrocytes were treated with APS *in vitro*, media from cultured chondrocytes contained significantly increased concentrations of IL-1Ra and IL-10 (19). Additionally, Woodell-May et al. showed that APS contained higher concentrations of growth factors, including a 6.8-fold increase in platelet-derived growth factor (PDGF) AB, a 2.7-fold increase in VEGF and a 5.1-fold increase in epidermal growth factor (EGF) in comparison to whole blood (20).

Although ACS products such as IRAP II™ (Arthrex, Naples, FL) and Orthokine® (Dechra Veterinary Products, Overland Park, KS) are used commonly in joints, there has been a widely held belief among equine clinicians that these products should not be used in tendon, ligament, bursa, and sheath injuries; however, this has not been validated. In a recent study, horses with SDFT tendinopathy were treated with ACS and compared to saline control treated limbs. They found that ACS treated tendons had increased type 1 collagen content, which may translate to increased tensile strength of the healed tissue (21). APS has been used anecdotally for intralesional treatment of tendon and ligament injuries, but its use in these injuries has never been studied *in vivo*. This combination orthobiologic may prove to be quite advantageous due to its combination of concentrated platelets, IL-1ra, and IL-10.

The objective of this study was to evaluate the effect of APS on tendon healing in an equine collagenase-induced superficial digital flexor tendonitis model. We hypothesized that a single intralesional injection of APS 7 days following collagenase induction of superficial digital flexor tendonitis would result in superior structural and biomechanical healing in comparison to placebo treatment.

## Materials and Methods

### Animals

Eight systemically healthy adult Thoroughbred horses between the ages of 3 and 8 years (median age = 5 years) were enrolled in the study. These horses had no history of previous superficial digital flexor tendinopathy. Additionally, prior to the start of the study, an ultrasonographic examination of the superficial digital flexor tendon was performed bilaterally to ensure that there was no evidence of previous superficial digital flexor tendinopathy. All animal procedures were approved by the Institutional Laboratory Animal Care and Use Committee at the University of Pennsylvania.

### Induction of Tendonitis

Superficial digital flexor tendonitis was induced in both forelimbs using collagenase injection as previously described by Watts et al. (22) with some slight modifications. Briefly, horses were sedated with 0.4 mg/kg xylazine, 0.01 ug/kg detomidine, and 0.04 mg/kg acepromazine intravenously. The palmar metacarpus was clipped and aseptically prepared, and local anesthesia was achieved with subcutaneous infiltration of 2% mepivacaine in a line block on the palmar aspect of the proximal metacarpus. At 12 cm distal to the accessory carpal bone (DACB), a stab incision was made through the skin at the site of needle entry. Using ultrasound guidance, A 17 g, 3.5-inch Tuohy Epidural Needle (Medline Industries, Inc. Northfield, IL) was used to create columnar separation of collagen fibers by advancing the needle from the palmar surface of the limb through skin and subcutaneous tissue incisions into the center of the SDFT. The entry point was 12 cm DACB, and the needle was advanced distally along the long axis of the SDFT until the tip of the needle was seen ultrasonographically at 18 cm DACB. The tip of the needle was withdrawn to 17 cm DACB, and as it was slowly withdrawn over a length of 1 cm, 1,000 units of sterilized bacterial collagenase type I (Sigma-Aldrich, St. Louis, MO) in 270 μL of sterile saline was injected. The needle and syringe were then withdrawn together. The limb was bandaged and this procedure was repeated on the contralateral limb.

Phenylbutazone was administered at 4.4 mg/kg intravenously following the completion of the procedure. Horses were box stall confined and received phenylbutazone at 2.2 mg/kg orally twice daily for 3 days, then 2.2 mg/kg orally once daily for 3 days. Forelimb bandages were maintained on both front limbs. Limb examinations, including assessment of swelling, heat, and pain on palpation were performed daily. Lameness (at the walk) and physical examination parameters were performed every 24 h.

### Intralesional Therapy

Seven days following induction of tendonitis, one forelimb was randomly assigned to receive an intralesional injection of APS (Pro-Stride™, Owl Manor, Huntington, IN) and the other a placebo treatment injection of saline. APS was prepared according to manufacturer's instructions and injected immediately after preparation (16). Briefly, 55 mL of blood was collected sterilely from the left jugular vein into a 60 mL syringe containing 5 mL of anticoagulant citrate dextrose, solution A (ACD-A). The blood was then processed into APS *via* centrifugation in the separation device followed by centrifugation in the concentration device. Local anesthesia of the limbs was achieved with subcutaneous infiltration of 2% mepivacaine in a line block on the palmar proximal metacarpus. The injection site was aseptically prepared, and 3 mL of APS or saline was injected into the lesion at 16 cm DACB using a 22 g, 1.5 inch needle under ultrasonographic guidance. Each limb was bandaged for 7 days following the procedure. Following intralesional therapy, horses were maintained on strict stall rest for 8 weeks. Beginning at 8 weeks following treatment injection, horses were hand walked for 5 min per day until study termination.

### Ultrasonographic Examination

Complete ultrasound examinations of both front SDFTs were performed immediately prior to induction of tendonitis (week −1) and then at week 0, 2, 4, 8, and 12 following treatment injection. Ultrasound-guided injection of saline or APS was performed at week 0 following ultrasonographic assessment of the SDFT. Ultrasonographic imaging was performed by a single ultrasonographer (C.U.) using an Aplio i700 ultrasound machine (Canon, Tustin, CA) with a 5–18 MHz linear probe and a stand-off. Longitudinal and transverse images were obtained from 8 cm distal to the accessory carpal bone (DACB) to 26 cm DACB at 2 cm intervals using standardized imaging settings. Quantitative measurements included tendon cross-sectional area (TCSA) and lesion cross-sectional area (LCSA). Lesion-cross sectional area was divided by TCSA to calculate the relative cross- sectional area of the lesion (%CSA). Ultrasound images were retrospectively assessed by 2 blinded observers (C.U. and V.B.R.) using a semi-quantitative scoring system adapted from Watts et al. (22). Tendons were evaluated for tendon echogenicity (0 = mostly normal; 1 = mostly echogenic; 2 = 50% echogenic and 50% anechoic; 3 = mostly anechoic), off-incidence tendon echogenicity (0 = mostly normal; 1 = mostly echogenic; 2 = 50% echogenic and 50% anechoic; 3 = mostly anechoic), linear fiber pattern (0 = 75–100% parallel fibers; 1 = 50–74% parallel fibers; 2 = 25–49% parallel fibers; 3 = 0–24% parallel fibers), and peritendinous edema (0 = none; 1 = minimal; 2 = moderate; 3 = severe). For statistical analysis, cross-sectional areas and scores were summed for the maximal injury zone (MIZ) from 14 to 18 cm DACB.

### Tissue Harvest

Twelve weeks following intralesional injection, horses were subjected to euthanasia *via* pentobarbital overdose. Forelimbs were dissected under RNAase-free conditions and the SDFT from 8 to 26 cm DACB was collected. The SDFT was then sectioned into quarters such that each $\frac{1}{4}$ had a representative sample of the lesion. The full length of the collected tendon was submitted for biomechanical testing. For histopathology, gene expression and biochemical analysis, 2 cm sections of SDFT were collected from 15 to 17 cm DACB. A similar sized section of unaffected (i.e., “normal”) tendon was collected at 12 cm DACB.

### Biomechanical Testing

One quarter of each SDFT from 8 to 26 cm DACB was tested. Biomechanical testing was performed on longitudinal slices of tendon samples. Samples were thawed at room temperature for ~60 min on the day of testing. A custom laser device (23) was used to measure mid-substance cross sectional area spanning 20 mm centered at the injury. A total of 10 measurements were taken with the final area calculated as the average of the 10 measured values. Hydration was maintained during all stages of test preparation. Each sample was tested as received and no further manipulations were performed. The termini of each sample were clamped in custom grips designed to ensure proper traction and minimize slippage. The grip-tendon-grip construct was then loaded on to an Instron materials testing machine (ElectroPuls E3000, Instron Inc., Norwood, MA) equipped with a 5000 N uniaxial load cell (Dynacell, Instron Inc., Norwood, MA). The loading protocol was initiated with a preload to 5 N, 10 cycles of preconditioning with an amplitude of 50 N at 1 Hz, immediately followed by a quasistatic ramp-to-failure at 0.1 mm/s. A gauge length image was taken during the preload stage to digitally calculate the gauge length. Every test was recorded to determine the proper mode of failure. All data including time (s), displacement (mm), and load (N), were recorded at 100 Hz. Stiffness (N/mm) was determined from the linear region of the resultant load-displacement curve. The load and displacement values were normalized by cross sectional area and gauge length, respectively, to obtain the stress-strain curves. The elastic modulus (MPa) was determined as the slope of the linear region from the stress-strain plots. Other parameters determined were failure load (N) and failure stress (MPa).

### Histopathology

Longitudinal tendon sections were fixed in 10% formalin, embedded in paraffin, sectioned and stained with Hematoxylin and Eosin (H&E), Masson's trichrome (MTC) and Picrosirius red (PSR) prior to examination under white light (H&E, MTC, PSR) and polarized light microscopy (PSR). Sections were scored by a blinded board-certified pathologist (J.B.E.), using a semi-quantitative scoring system as previously described (22). Entire tendon sections were first evaluated under low and high power followed by scoring for tenocyte linearity, tenocyte density, hemorrhage, neovascularization, perivascular cuffing, collagen fiber linearity, collagen fiber uniformity, epitenon thickening and polarized light crimping. All parameters were score from 1 (normal architecture/no lesions) to 4 (severe).

### Gene Expression

Tendon samples collected for gene expression analysis were snap frozen in liquid nitrogen and biopulverized in liquid nitrogen using a multi-sample stainless steel biopulverizer (BioSpec Products, Inc., Bartlesville, OK). RNA was isolated using a lysis reagent (QIAzol Lysis Reagent, Qiagen, Germantown, MD) and a commercially available RNA extraction kit (Qiagen RNeasy Tissue Kit, Qiagen, Germantown, MD). RNA concentration and purity were quantified using a UV microspectrophotometer (NanoDrop™ One, ThermoFisher Scientific, Waltham, MA). Complementary DNA was prepared using a High Capacity cDNA Reverse Transcription kit (ThermoFisher Scientific, Waltham, MA) and an Eppendorf master cycler (Hamburg, Germany). Real-time quantitative PCR was performed using TaqMan™ Master mix and the Applied Biosystems™ QuantStudio™ 6 Flex Real-Time PCR System (Applied Biosystems, Foster City, CA). Expression of *MMP1* (matrix metalloproteinase 1), *MMP3* (matrix metalloproteinase 3), *MMP13* (matrix metalloproteinase 13), *ADAMTS4* (a disintegrin and metalloproteinase with thrombospondin motifs 4), *COL1A1* (collagen type I), *COL3A1* (collagen type III), *SCX* (scleraxis), *TNMD* (tenomodulin), *TNC* (tenascin-C), *DCN* (decorin), and *COMP* (cartilage oligomatrix matrix protein) were quantified.

Primers and probes for *18S, MMP3*, and *TNC* were designed using NCBI Primer-BLAST and Integrated DNA Technologies (IDT) PrimerQuest Tool software and synthesized by IDT (Coralville, IA). The primers and probes for *18S, MMP3*, and *TNC* were as follows: *18S*, forward, 5′- GCCGCTAGAGGTGAAATTCT-3′, reverse, 5′-TCGGAACTACGACGGTATCT−3′, probe, 5′-AAGACGGACCAGAGCGAAAGCAT-3′; *MMP3*, forward, 5′-ATGGACCTTCTTCAGGACTACC-3′, reverse, 5′GACCGACATCAGGAACTCCG-3′, probe, 5′-TGACACTGTGGAGGTGATGCACAA-3′; and *TNC*, forward 5′-GCCCCTGGCTGAAATCG-3′, reverse 5′-CGGTCACCTGGCAGATCTT-3′, probe, 5′-CGGCATCGAGCTCACCTATGGTGTC-3′. Primers and probes for *MMP1, MMP13, ADAMTS4, COL1A1, COL3A1, SCX, TNMD, DCN*, and *COMP* were obtained from ThermoFisher Scientific's proprietary equine-specific gene expression assay database (ARCE46U) (Supplementary Table 1). All samples were run in triplicate using *18S* as a reference gene. The cycle threshold (CT) values for triplicates were averaged and data were analyzed using the ΔCt method where fold change is expressed as 2^−Δ*ΔCt*^ using unaffected tendon as the calibrator.

### Biochemical Analysis

Tendon samples collected for biochemical assays were snap frozen, biopulverized in liquid nitrogen and lyophilized. Total glycosaminoglycan (GAG) was quantified using the dimethylmethylene blue (DMMB) assay with chondroitin-4 sulfate used to establish a standard curve and optical density determined at 525 nm (24). Total DNA content was determined using papain digested tissues that were incubated for 24 h at 65°C and then mixed with Hoescht (Sigma-Aldrich, St. Louis, MO) for quantification by fluorometric assay at 348 nm.

### Statistical Analysis

Power analysis was conducted with a two-sample paired-means. Preliminary data used to perform power analysis was from Geburek et al. (11). Specifically, data from the elasticity modulus comparing tendonitis treated with mesenchymal stem cells and serum or serum alone with normal tendon. The analysis with eight animals resulted in a power of 0.99, sufficient to determine statistical significance with the primary outcome measure. Data were analyzed using JMP Pro 14 (SAS, Cary, NC). A mixed effects model was used for analysis of all outcome variables with horse as a random effect and treatment and day (for repeated measures) as fixed effects with an interaction term for day and treatment. Day was treated as a categorical variable to allow for the non-linear effect of time. Multiple comparisons were made with a Tukey's *post-hoc* test. Significance was set at *p* ≤ 0.05.

## Results

### Ultrasonography

**Figure 1:**
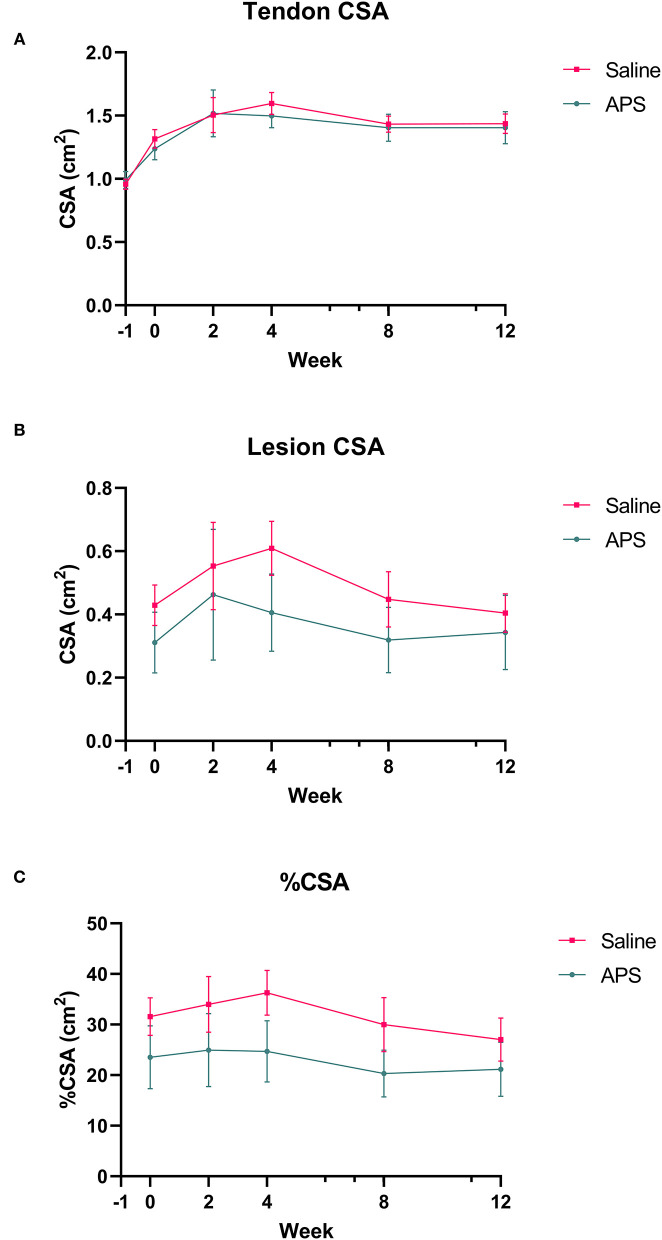
Mean (±SEM) cross-sectional area (CSA) of the **(A)** tendon and **(B)** lesion at the maximal injury zone (14–18 cm DACB) at 0, 2, 4, 8, and 12 weeks following intra-lesional injection of saline or APS. Relative cross sectional area of the lesion (%CSA) occupied by the lesion is shown in **(C)** (*n* = 8).

**Figure 2:**
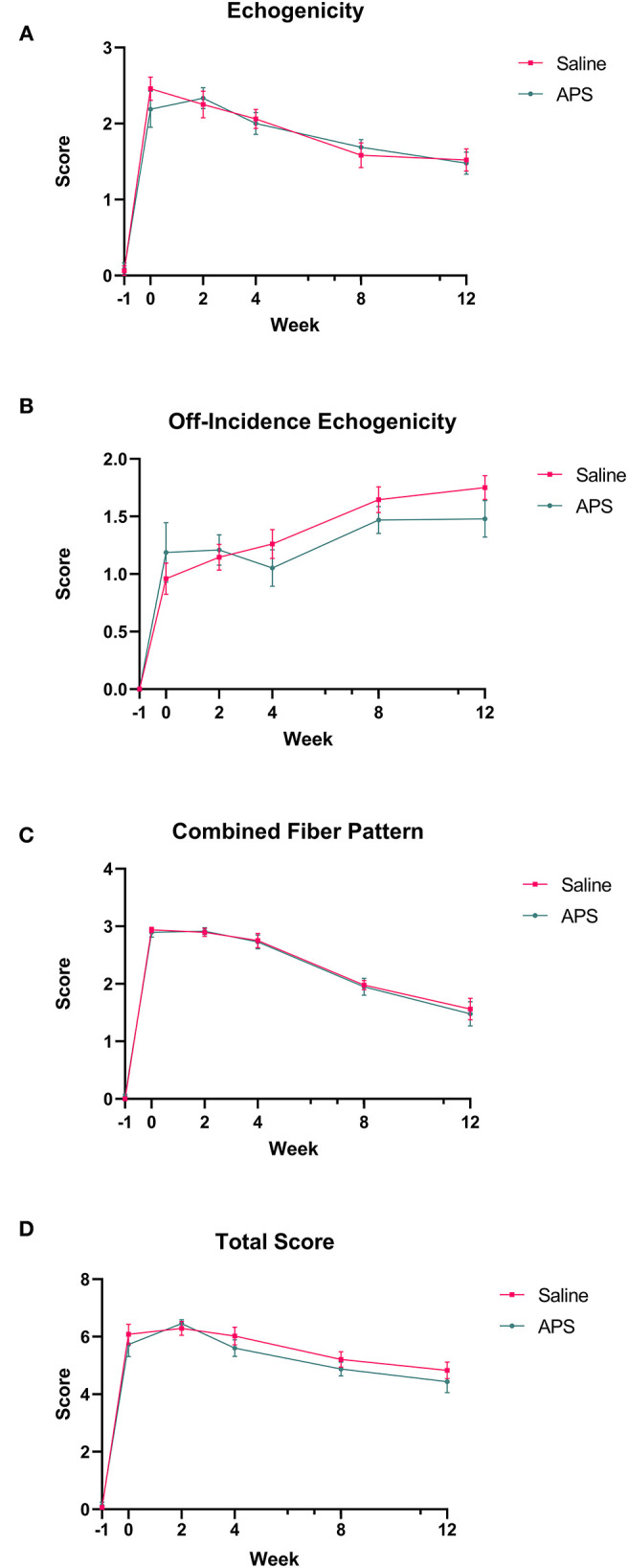
Mean (±SEM) scores for **(A)** tendon echogenicity, **(B)** off-incidence tendon echogenicity, **(C)** tendon fiber pattern, and **(D)** total ultrasonographic score at the maximal injury zone (14–18 cm DACB) at 0, 2, 4, 8, and 12 weeks following intra-lesional injection of saline or APS (*n* = 8).

Collagenase injection resulted in centralized core lesions in all limbs. In both saline and APS treated tendons, at the maximal injury zone TCSA, LCSA and %CSA increased linearly until week 2 (APS) or week 4 (saline). After week 4, the TCSA plateaued or decreased slightly, while LCSA decreased from week 4 to 12 (Figure 1). For APS treated tendon, LCSA and %CSA were lower than in saline treated tendon at all time points, however there were no statistically significant differences. At the MIZ, ultrasonographic scores including tendon echogenicity, off-incidence tendon echogenicity, linear fiber pattern, and peritendinous edema were not significantly different between APS and saline treated tendons at any time point (Figure 2).

### Biomechanical Testing

**Figure 3:**
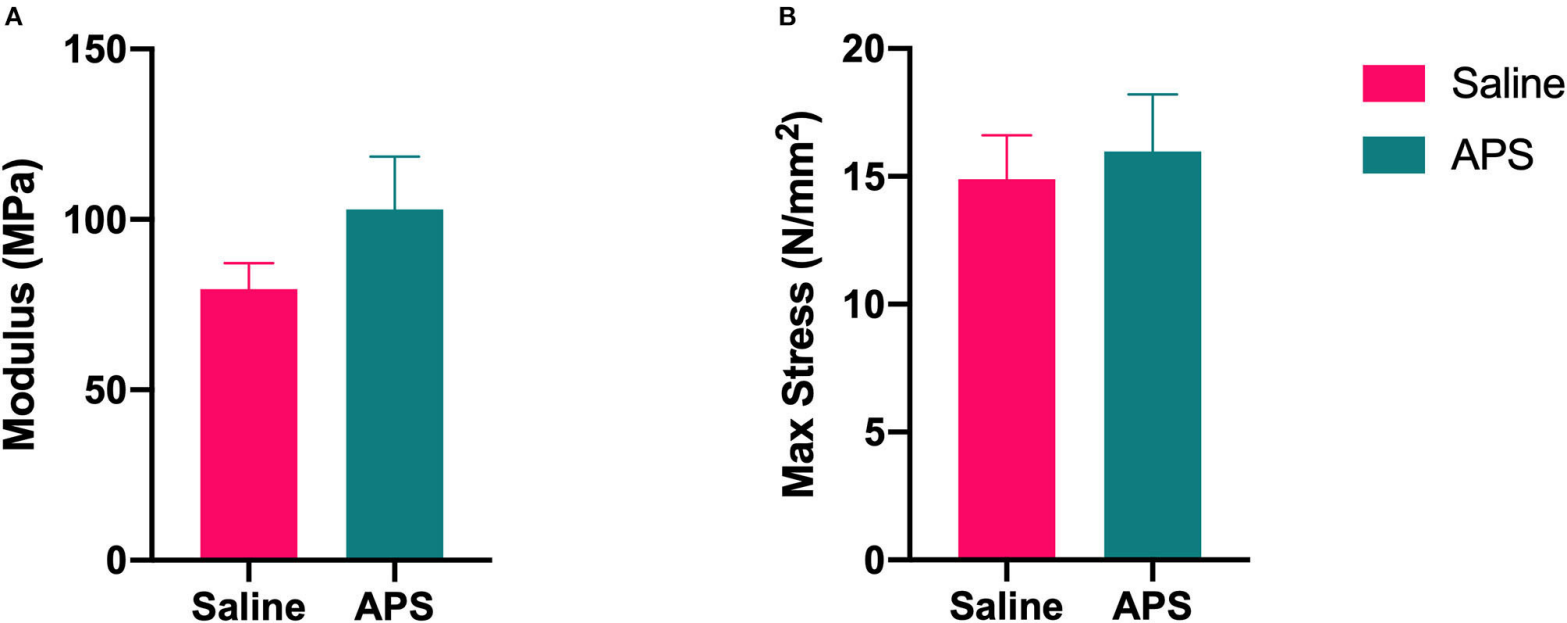
Biomechanical testing results for **(A)** elastic modulus (mean ± SEM) and **(B)** max stress at failure (mean ± SEM) for APS treated and saline treated tendon (*n* = 8).

The mean (±SEM) cross sectional area of tendon samples tested was 57.96 ± 1.08 mm^2^, and the mean (±SEM) gauge length was 56.05 ± 0.68 mm. In the saline treated tendon samples, there were three midsubstance failures, four samples that failed at the grip, and one that failed at the grip and at the injury site. In APS treated tendon samples, one failed at the injury, 6 failed at the grips, and one failed in the midsubstance and at the grip. Mean (±SEM) modulus was higher for APS treated tendons (102.94 ± 5.14 MPa) than saline treated tendons (79.55 ± 2.53 MPa), but this difference was not significantly different. No significant differences were detected for cross-sectional area, failure load, stiffness, or failure stress between APS treated and saline treated tendons (Figure 3).

### Histopathology

**Figure 4:**
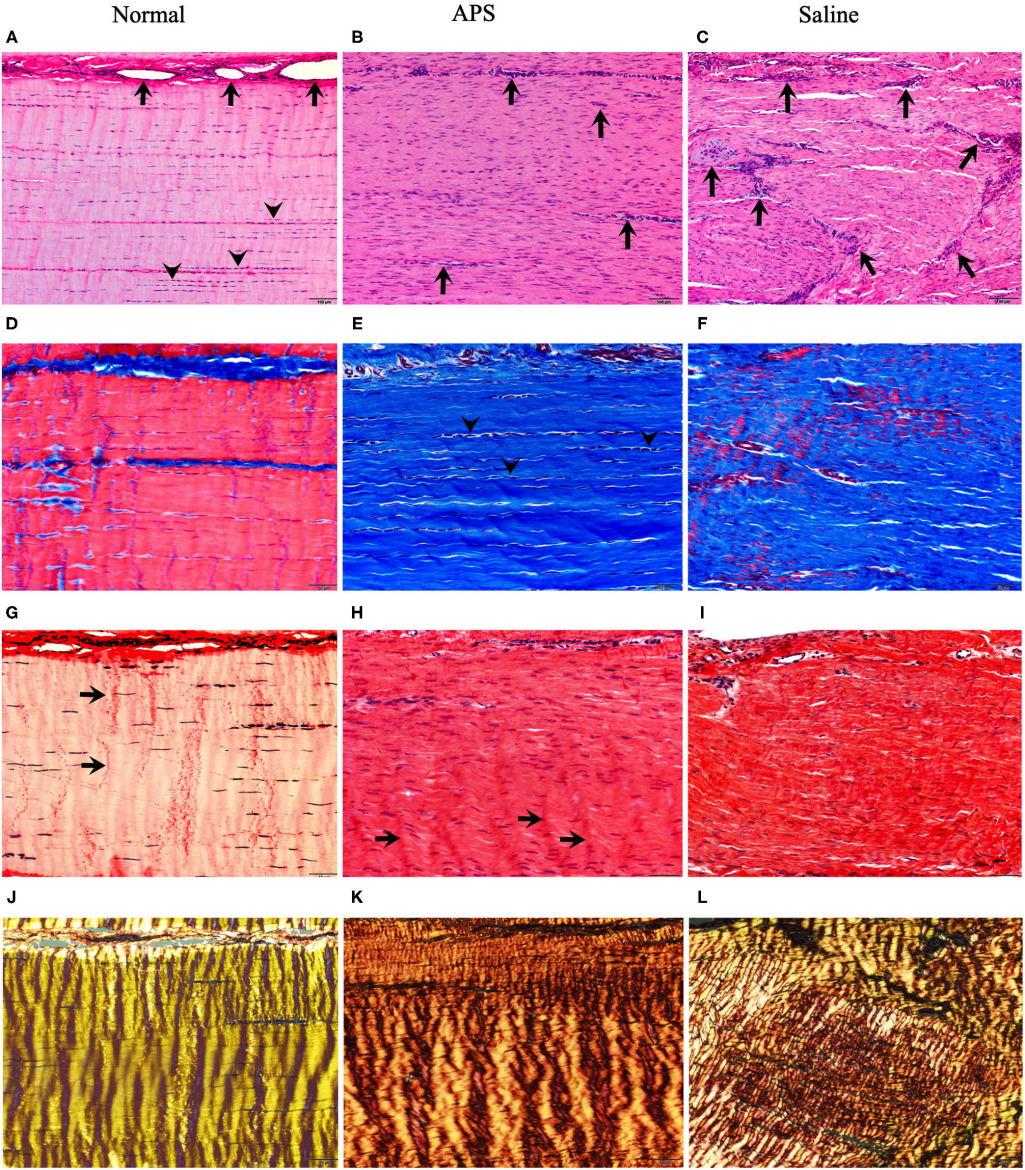
Photomicrographs stained with H&E **(A–C)** Masson's trichrome (MTC) **(D–F)** and Picrosirius red examined under unpolarized (PSR) **(G–I)** and polarized (PSR-P) **(J–L)** light for normal (**left panels**), APS treated (**middle panels**) and saline treated (**right panels**) tendons. H&E stains show in normal tendons **(A)** blood vessels (**arrows**) are limited to endotendinous septae with multifocal nuclear rowing (**arrowheads**), compatible with mild degeneration. In contrast, APS **(B)** and saline-treated **(C)** tendons contain increased numbers of small caliber blood vessels randomly interspersed among regenerating tendon fibers which have increased, uniformly dispersed numbers of tenocyte nuclei (10 × magnification; bars = 100 μm). MTC stains of normal tendons **(D)** show red staining of well-defined linear arrays of tendon fibers compared to APS **(E)** and saline **(F)** treated tendons that show blue staining of tendon fibers, but APS treated tendons have increased parallel alignment of tendon fibers with linearly-aligned tenocyte nuclei (**arrowheads**) compared to saline treated tendons (20 × magnification; bars = 50 μm). PSR stained sections of normal tendons **(G)** show uniform crimp (**arrows**) with few numbers of thin, wavy tenocyte; APS **(H)** and saline **(I)** treated tendons have increased numbers of more blunted oval nuclei with development of crimp (**arrows**) in APS treated tendons (20 × magnification; bars = 50 μm). PSR-P images highlight regular, well-developed crimp and bright yellow refractility in normal tendons **(J)**; APS-treated tendons **(K)** show further developed and regular periodic crimp with yellow-orange refractility compared to saline treated tendons **(L)** that have coarsely interwoven broad streams of fibers with a combination of short yellow-orange to yellow-green refractility (20 × magnification; bars = 50 μm).

**Figure 5:**
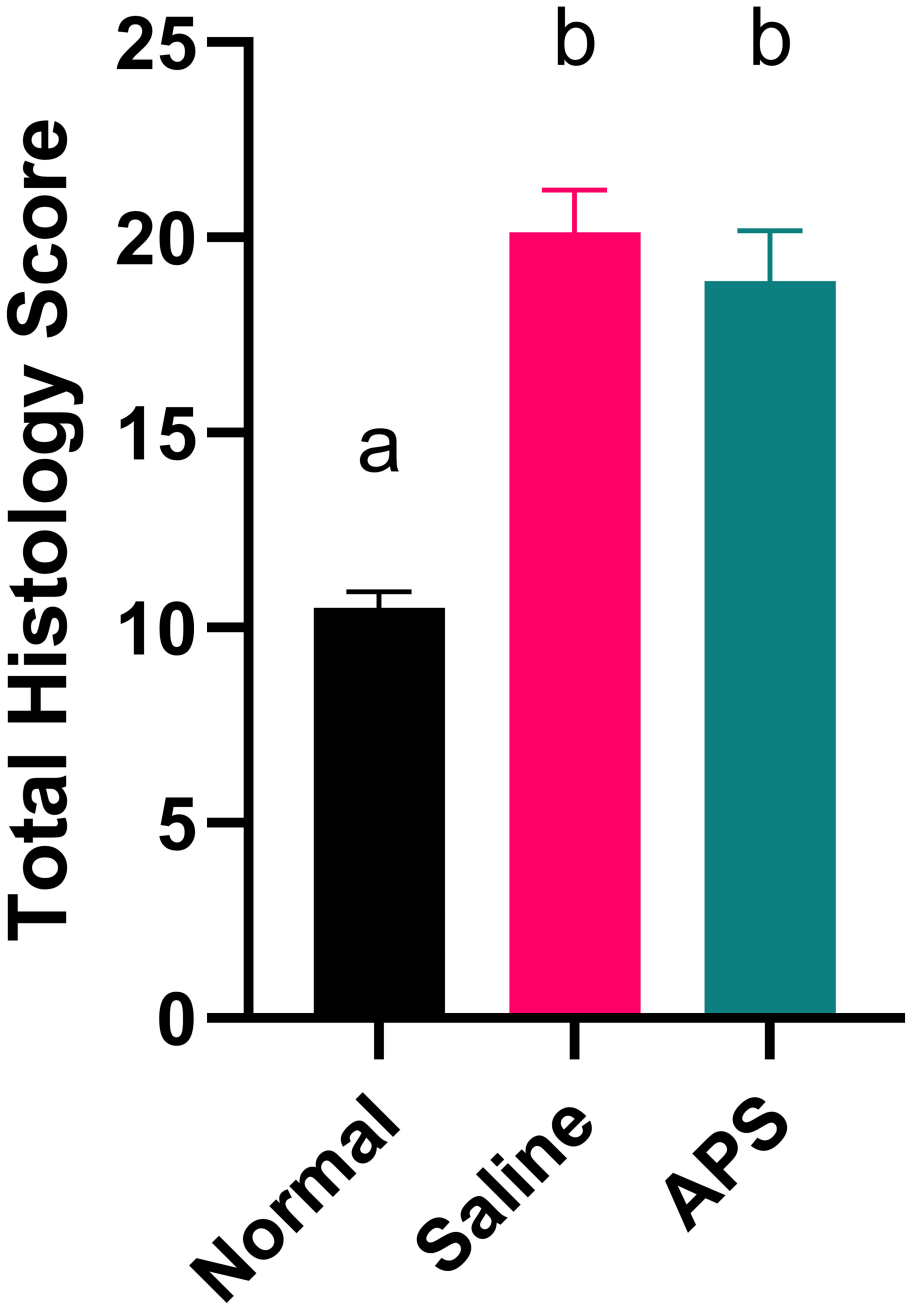
Total histologic score (mean ± SEM) at 16 cm DACB 12-weeks following intra-lesional injection of saline or APS. Total histologic scores for normal tendon are included for reference. Total scores represent the sum of histologic scores for tenocyte linearity, tenocyte density, hemorrhage, neovascularization, perivascular cuffing, collagen fiber linearity, collagen fiber uniformity, epitenon thickening and polarized light crimping. Different letters denote significant differences between groups, *p* less than or equal to 0.05 (*n* = 8).

Mean (± SEM) total histology score for tendon samples evaluated for tenocyte shape, tenocyte density, hemorrhage, neovascularization, inflammatory cell infiltrates, collagen fiber linearity, collagen fiber uniformity, epitenon thickening and polarized light crimping were significantly lower for normal tendons (10.50 ± 0.39) compared to APS treated tendons (18.88 ± 1.22) or saline treated tendons (20.14 ± 0.30) (Table 1 and Figure 4). Scores of individual parameters were also significantly lower in normal tendons compared to APS treated or saline treated tendons (Figure 5). Tenocyte shape and density, inflammatory cell infiltrate, collagen fiber linearity and uniformity, polarized collagen fiber crimp and epitenon thickening were lower for APS-treated tendons compared to saline-treated tendons, however, no statistically significant differences were noted (Table 1).

**Table 1:** Mean scores (±SEM) of individual parameters for tenocyte shape, tenocyte density, hemorrhage, neovascularization, inflammatory cell infiltrates, collagen fiber linearity, collagen fiber uniformity, polarized light crimping, and epitenon thickening for normal tendons, saline treated tendons, and APS treated tendons.

	**Tendon cell shape**	**Tendon cell density**	**Free hemo-rrhage**	**Neo-vasculature**	**Inflam-matory cell infiltrate**	**Collagen fiber linearity**	**Collagen fiber uniformity**	**Polarized collagen fiber crimp**	**Epitenon thickening**	**Total score**
Normal	1.63 (±0.17)^a^	2.00 (±0.00)^a^	1.00 (±0.00)^a^	1.00 (±0.00)^a^	1.00 (±0.00)^a^	1.00 (±0.00)^a^	1.63 (±0.17)^a^	1.25 (±0.15)^a^	1.00 (±0.00)^a^	10.50 (±0.39)^a^
Saline	2.71 (±0.05)^b^	3.29 (±0.08)^b^	1.43 (±0.06)^ab^	2.86 (±0.09)^b^	1.71 (±0.05)^b^	2.57 (±0.06)^b^	2.86 (±0.04)^b^	2.71 (±0.08)^b^	1.57 (±0.10)^b^	20.14 (±0.30) ^b^
APS	2.38 (±0.17)^b^	3.13 (±0.21)^b^	1.63 (±0.17)^b^	3.00 (±0.25)^b^	1.63 (±0.17)^b^	2.25 (±0.29)^b^	2.50 (±0.18)^b^	2.38 (±0.30)^b^	1.13 (±0.12)^b^	18.88 (±1.22)^b^

*Different letters denote significant differences between groups, p ≤ 0.05*.

### Gene Expression

**Figure 6:**
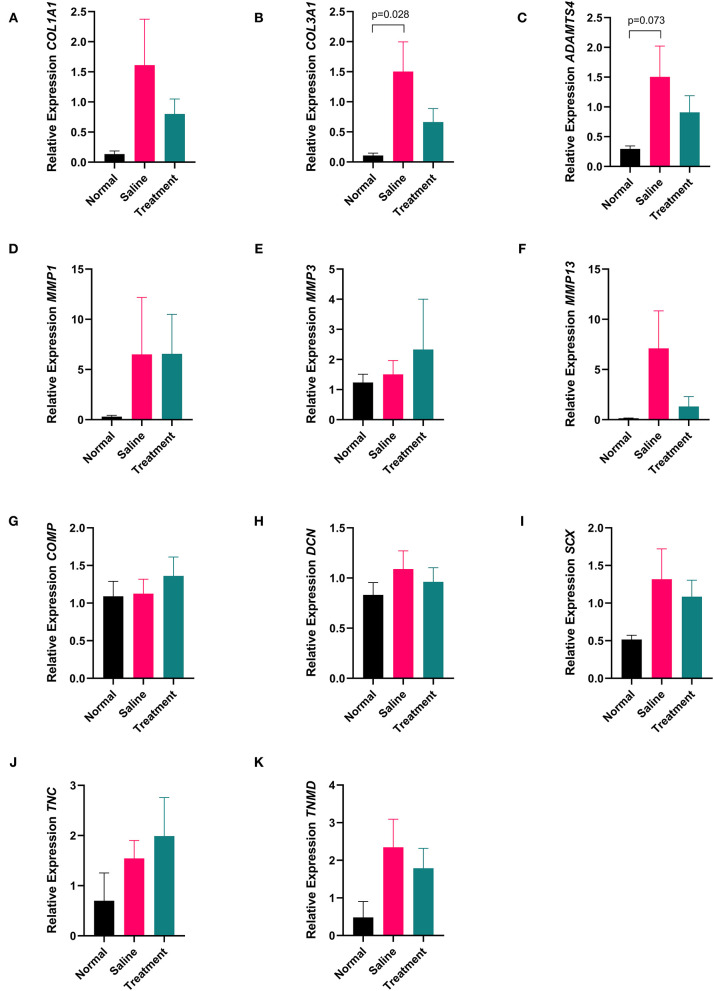
Quantitative PCR data showing expression **(A)** *COL1A1*, **(B)** *COL3A1*, **(C)** *ADAMTS4*, **(D)** *MMP1*, **(E)** *MMP3*, **(F)** *MMP13*, **(G)** *COMP*, **(H)** *DCN*, **(I)** *SCX*, **(J)** *TNC*, and **(K)** *TNMD* from tendon collected 16 cm DACB 12-weeks following intra-lesional injection with saline or APS. Mean ± SEM for 8 horses are shown. Data were analyzed using the ΔCt method where fold change is expressed as 2^−Δ*ΔCt*^ using *18S* as a reference gene and normal tendon as the calibrator (*n* = 8).

Tendon samples from the point of maximal injury in APS treated, saline treated, and normal tendon were analyzed for expression of *MMP1, MMP3, MMP13, ADAMTS4, COL1A1, COL3A1, SCX, TNMD, TNC, DCN*, and *COMP* using real time quantitative PCR. Mean (±SEM) fold change of *COL3A1* expression was significantly increased (p=0.028) in saline treated tendons (22.32 ± 6.88) compared to normal tendons (1.58 ± 0.54). In contrast, *COL3A1* expression in APS treated tendons (9.86 ± 3.16-fold change) was not significantly different from normal tendons. There was a trend toward increased expression of ADAMTS4 in saline treated tendon compared to normal tendon (*p* = 0.073). No other significant differences in gene expression were noted (Figure 6).

### Biochemical Analysis

**Figure 7:**
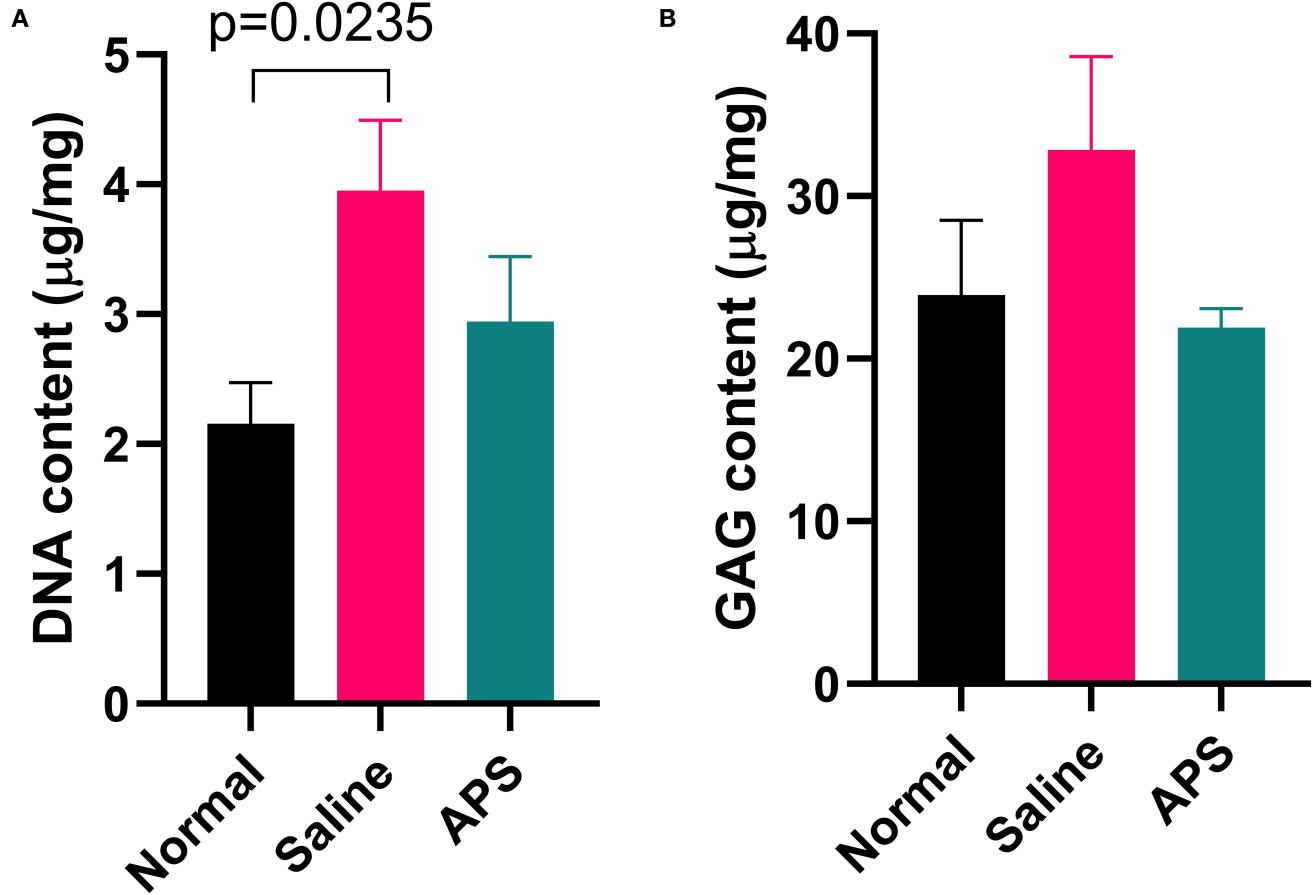
Mean (± SEM) content of **(A)** DNA and **(B)** GAG of normal, saline treated, and APS treated tendon (*n* = 8).

Tendon samples from the point of maximal injury in APS treated, saline treated, and normal tendon were analyzed for total DNA and total GAG content. Mean (±SEM) total DNA content was significantly higher (*p* = 0.024) in saline treated tendons (3.95 ± 0.51 μg/mg), compared to normal tendons (2.16 ± 0.30 μg/mg), whereas total DNA content was not significantly different between APS treated tendon (2.94 ± 0.47 μg/mg), and normal tendon (Figure 7). Total GAG content was not significantly different between APS treated, (21.90 ± 1.11 μg/mg), saline treated (32.86 ± 5.35 μg/mg), and normal tendons (23.93 ± 4.29 μg/mg) (Figure 7).

## Discussion

APS may improve tendon healing by combining the beneficial effects of ACS, which contains concentrated interleukin-1 receptor antagonist (IL-1ra) and interleukin-10 (IL-10), with the beneficial effects of PRP, which contains a multitude of growth factors and anti-inflammatory cytokines (16, 17, 20). While ACS and PRP have been shown to have beneficial effects on tendon healing, the effects of APS on tendon healing have not been studied (6, 9, 25). In our study, we found that treatment with APS following collagenase-induced SDFT tendonitis resulted in decreased expression of collagen type III in comparison to saline-treated tendons. We also found that total DNA content was not different between normal and APS treated tendons, while it was increased in saline treated tendons.

Collagen type III has been shown to be upregulated in equine tendonitis models, especially during the early phases of healing, whereas collagen type I is typically upregulated in the later phases of healing (10, 26). Collagen type III has less elasticity than collagen type I, therefore increased collagen type III deposition is thought to result in inferior biomechanical healing (11, 27). Decreased collagen type III expression in APS treated tendons in our study may indicate superior healing. Although gene expression of *COL3A1* was increased in saline treated tendons in our study, we cannot definitively conclude this resulted in increased collagen type III content as the histochemical stains and grading scheme used were not able to definitively discern differences in collagen composition. Further quantitative analysis such as colorimetric histomorphometry, immunohistochemistry or western blot analysis would be needed to confirm and further characterize collagen type III content.

There was a trend (*p* = 0.073) toward upregulation of expression of ADAMTS4 in saline treated tendons in comparison to normal tendons. As a metalloproteinase and aggrecanase, ADAMTS4 has catabolic properties within tendon, and is associated with tendon-matrix turnover (12, 28, 29). In addition to acting as an aggrecanase, ADAMTS4 also acts to cleave non-proteoglycan components of the extracellular matrix, including COMP, fibromodulin, and decorin (30, 31). In human Achilles tendonitis, ADAMTS4 was found to be upregulated in ruptured tendons, in comparison to both normal and painful tendons (28). The role of ADAMTS4 in equine tendonitis is not well-elucidated. One study found that expression of ADAMTS4 decreased throughout the SDFT 6 weeks following transection of the tendon in the mid-metacarpal region; however, they also found that expression of ADAMTS4 was correlated with poorer collagen fiber alignment on histology (32). Another study found no difference in ADAMTS4 expression between MSC-treated and saline-treated control tendons following collagenase-induced tendonitis but did not quantify gene expression in unaffected tendon (12). Considering the role of ADAMTS4 in extracellular matrix turnover, increased expression may indicate ongoing tendon healing in saline treated tendons in our study.

In saline treated tendons, we found that DNA content was significantly increased, whereas DNA content was not different between APS treated and normal tendons. Increased DNA content is thought to be associated with ongoing healing processes in which high cellularity in the tendon is present (33). In other studies, DNA content has been found to be increased in abnormal tendon in both collagenase induced and surgically induced tendonitis models (11, 22). Although not statistically significant, total GAG content was higher in saline treated tendons, while it was similar in normal and APS treated tendons. Increased GAG content reflects high metabolism of tenocytes, and GAG is typically increased during early tendon healing. In human patellar tendonitis, increased GAG content is associated with greater tendon dysfunction (34). Our findings are similar to those of other models of tendonitis in the horse, where GAG content was higher in saline-treated tendons than in tendon treated with BM-MSCs or normal tendon (11, 14). Additionally, following transection of the SDFT in one study, GAG content increased throughout the affected tendon (32). Again, in our study increased DNA and increased GAG content in saline treated tendons may indicate ongoing healing processes in comparison to APS treated tendons.

Total histology scores were lower for APS treated tendons compared to saline treated tendons, although the difference was not statistically significant. Within categories, differences between saline treated and APS treated tendons were seen in tendon cell shape and density, collagen fiber linearity, collagen fiber uniformity, polarized collagen fiber crimp, and epitenon thickening. APS treated tendons had lower (i.e., less severe) scores in each of these categories.

During biomechanical testing, the elastic modulus of APS treated tendon was higher than saline treated tendon. Although this difference was not significant, this improvement could be clinically relevant in terms of tendon healing and strength. In a study by Geburek et al. (11), which had a similar biomechanical testing protocol, they tested normal tendon in addition to their study samples. The elastic modulus of normal tendon was 175 MPa, which is not considerably higher than the modulus of APS treated tendon in our study (103 MPa). Many of our tendon samples failed at the grips rather than mid-substance or at the injury, which may have resulted in a falsely decreased elastic modulus. As the tendons in this study were tested 13 weeks after collagenase-induced injury, they were not completely healed at the time of biomechanical testing. Further studies may elucidate whether the differences in biomechanical properties become more pronounced with longer healing times, and whether the increase in modulus in APS treated tendons correlates with greater resistance to re-injury.

There are several models described to study equine tendon injury *in vivo*, including injection of collagenolytic enzymes (12, 13, 15, 22, 26, 35–40), surgical induction of lesions (9, 11, 41–45) and radiofrequency coblation (46). In naturally occurring tendonitis, accumulated damage leads to an acute disruption of tendon fibers centrally followed by enzymatic degradation of normal tendon matrix (47, 48), thus the ideal model would mimic these conditions. While surgically created lesions can be created more uniformly, they often fail to exhibit the degree of inflammation reported of naturally occurring lesions and usually require general anesthesia to perform (42, 43). Inherent limitations of the collagenase tendonitis model include induction of lesions that may not have uniform size across all specimens. While these lesions are not biologically identical to those that arise spontaneously (i.e., naturally occurring disease), the creation of a central core lesion, enzymatic destruction of tendon matrix without disruption of the epitenon, and histologic evidence of inflammation was consistent in all cases in our study. Early reports of collagenase-induced tendonitis *via* direct injection of bacterial collagenase described expansive lesions extending to the epitenon causing peri-tendinous fibrosis and adhesions (36). However, recent improvements on this technique, which use a needle to cause columnar separation of fibers and create a physical defect before injection of collagenase, have resulted in more predictable lesions confined to the central core (22). Induction of tendonitis using collagenase more closely mimics the destruction of tendon fibrils by degradative enzymes, and subsequent changes in inflammation, acute swelling, tendon cross sectional area, and ultrasonographic appearance are similar to naturally occurring disease (36, 38). Additionally, collagenase injection can be performed in the standing horse, minimizing the distress of animal subjects and avoiding the risk of general anesthesia. While the effects of bacterial collagenase on the efficacy of APS is unknown, intralesional treatment 5–7 days following collagenase injection has been reported in several other studies (10, 12, 15, 39).

There were also potential artifacts of processing for histology. Lesion severity scores may have been confounded by either inconsistency in targeting the central region of each tendon lesion during the trimming process or regional variation in degree or extent of healing within each lesion. Moreover, the use of this semi-quantitative grading scheme may not be sensitive enough to identify potentially subtle differences between groups. As mentioned above, development and application of a morphometrical/colorimetrical scheme standardized to equine tendons that could include immunohistochemical stains may improve the sensitive of detecting differences between the groups in this model.

Finally, the 12-week study period is relatively short in comparison to the duration of the normal tendon healing processes in the horse. However, terminating the study at 12 weeks allowed us to determine the effects of APS treatment during the fibroplasia stage of healing. A previous study investigating temporal changes following collagenase induction of tendonitis showed no differences in biochemical analysis and minimal differences in histological healing between 8 and 24 weeks post-injury (26), suggesting a slow progression of healing after 8 weeks. A study investigating the effects of APS on naturally occurring tendonitis with long term follow up at 8–12 months would also be beneficial to determine if this treatment reduces re-injury rate or allows for a faster return to work.

### Conclusion

In an equine collagenase-induced tendonitis model, intralesional treatment with APS resulted in decreased collagen type III gene expression and decreased DNA content in comparison to saline treated tendons. This may translate to improved tendon healing, however further studies are needed to determine the effects of APS in naturally-occurring tendonitis, and to determine if APS offers an advantage over other orthobiologics such as PRP.

## Data Availability Statement

The raw data supporting the conclusions of this article will be made available by the authors, without undue reservation.

## Ethics Statement

The animal study was reviewed and approved by The Penn Office of Animal Welfare—Institutional Animal Care and Use Committee.

## Author Contributions

AG contributed to study design, assisted with data collection, analysis, and interpretation. Prepared the manuscript and approved the final manuscript. CU performed ultrasonographic examinations, collected ultrasound data, scored ultrasound exams, and approved the final manuscript. RL assisted with study design and performed the biochemical and gene expression analysis, and approved the final manuscript. KE assisted with biochemical and gene expression analysis and approved the final manuscript. VR scored ultrasound exams and approved the final manuscript. SS performed the biomechanical testing and assisted with interpretation of results and approved the final manuscript. RM and WK assisted with study design and approved the final manuscript. JE assisted with study design, prepared histologic samples and performed histologic scoring, prepared histology images for manuscript, and approved the final manuscript. KO assisted with study design, oversaw all aspects of data collection, performed data analysis and interpretation, reviewed manuscript, and approved the final version. All authors contributed to the article and approved the submitted version.

## Conflict of Interest

WK is the Director of Research and Development at Owl Manor, who's product was investigated in this study. The remaining authors declare that the research was conducted in the absence of any commercial or financial relationships that could be construed as a potential conflict of interest.
