# Supplementary material for: Evaluation of Autologous Protein Solution Injection for Treatment of Superficial Digital Flexor Tendonitis in an Equine Model
Source: Front Vet Sci. 2021 Jul 5;8:697551. doi: 10.3389/fvets.2021.697551 (PMC8287003; doi:10.3389/fvets.2021.697551)
Supplement: Supplementary file 1 [file Table_1.DOCX]

| **Gene symbol** | **Assay ID** | **GenBank Accession Number** | **Amplicon length (bp)** |
| --- | --- | --- | --- |
| *ADAMTS4* | Ec03469176_m1 | AF368321  EU025848 | 72 |
| *COL1A1* | Ec03469676_m1 | AF034691 | 154 |
| *COL3A1* | Ec03469743_m1 | AF117954 | 62 |
| *COMP* | Ec03468062_m1 | AF325902 | 54 |
| *DCN* | Ec03468475_m1 | AF038127 | 62 |
| *MMP1* | Ec03468020_m1 | AF148882 | 70 |
| *MMP13* | Ec03467796_m1 | AF034087 | 119 |
| *SCX* | Ec03818452_s1 | AB254030 | 76 |
| *TNMD* | Ec03467883_m1 | AB059407 | 72 |
